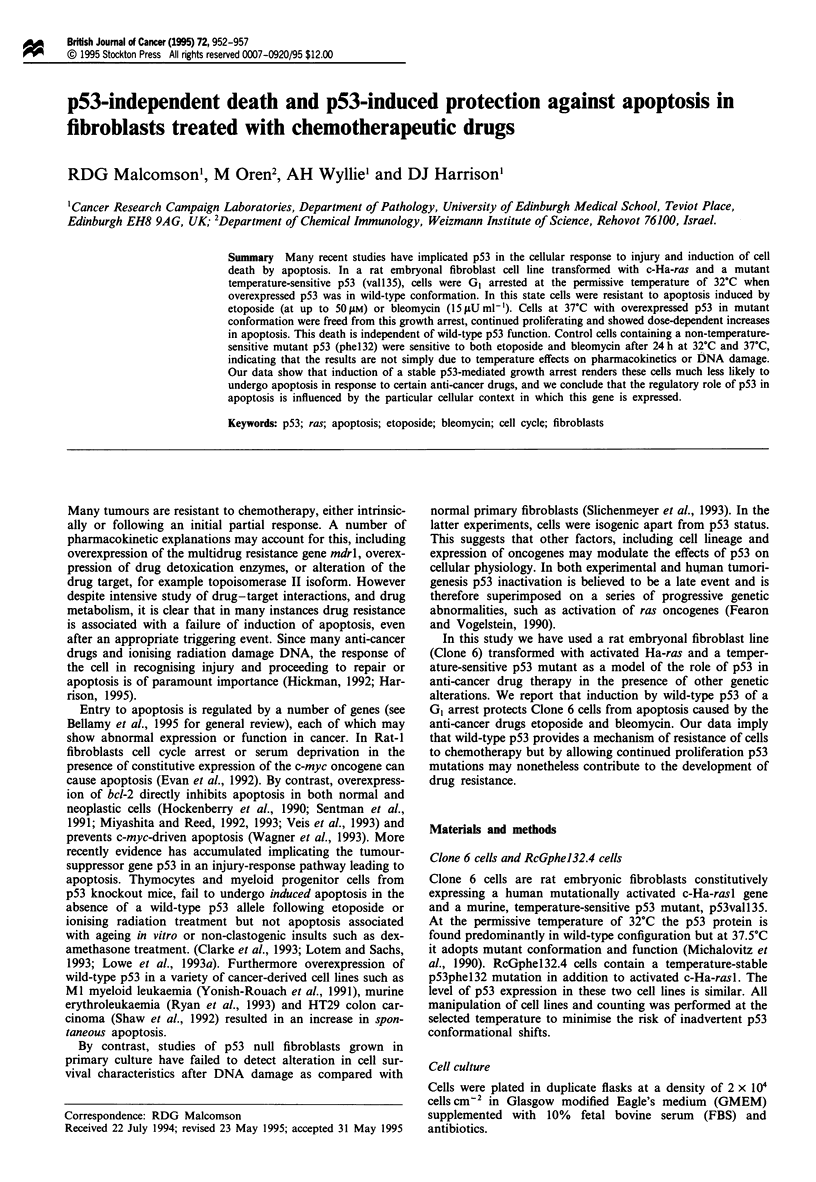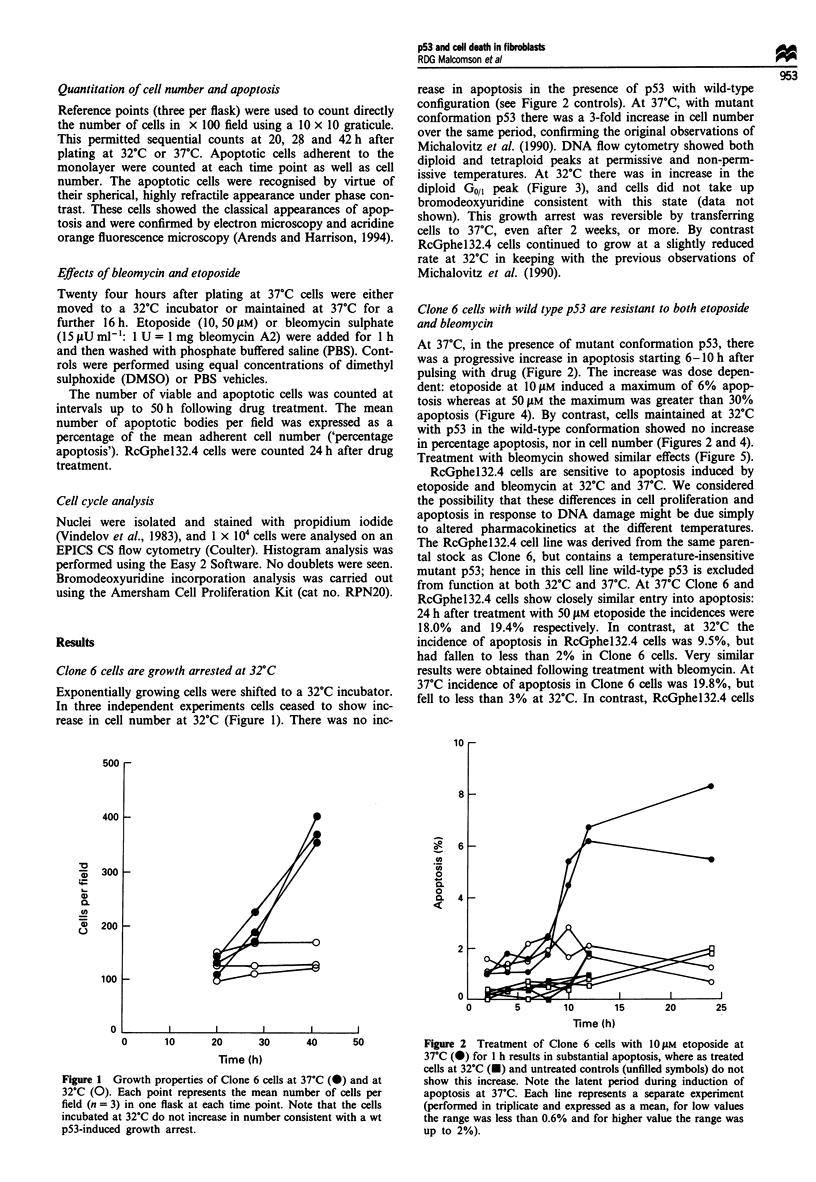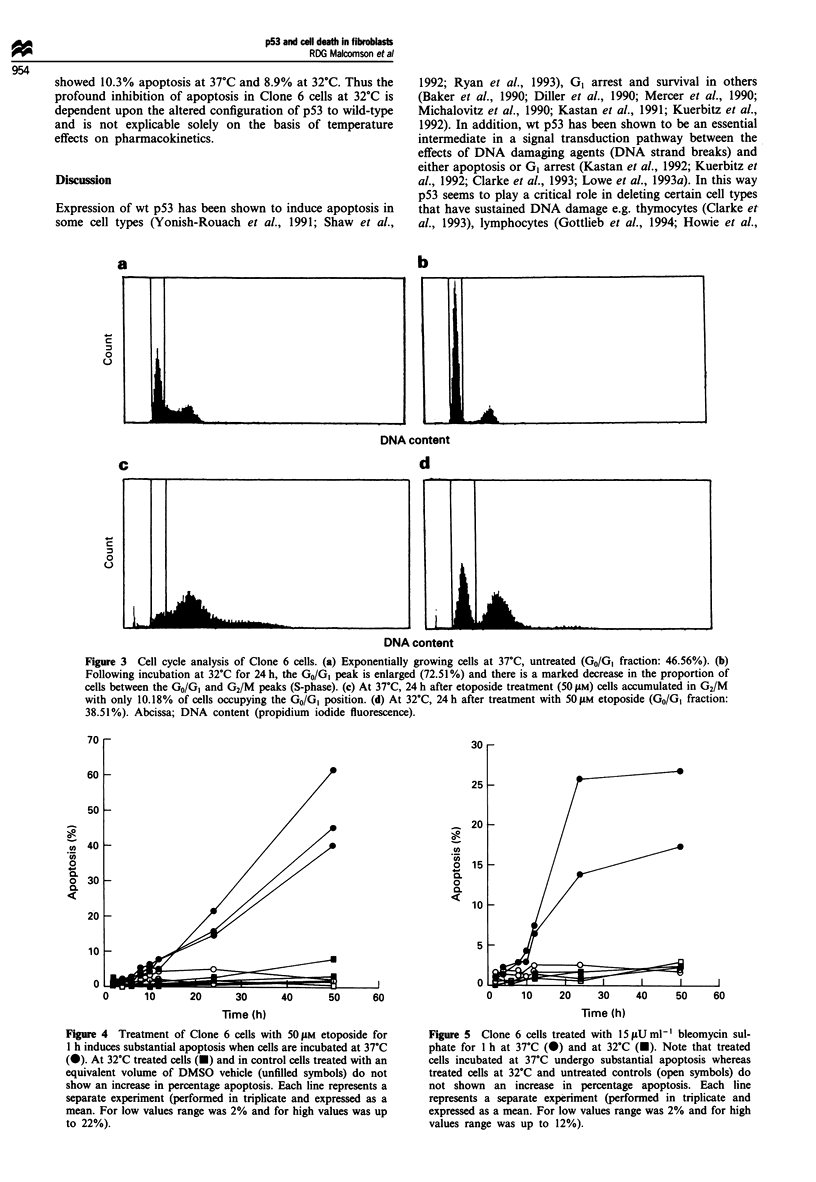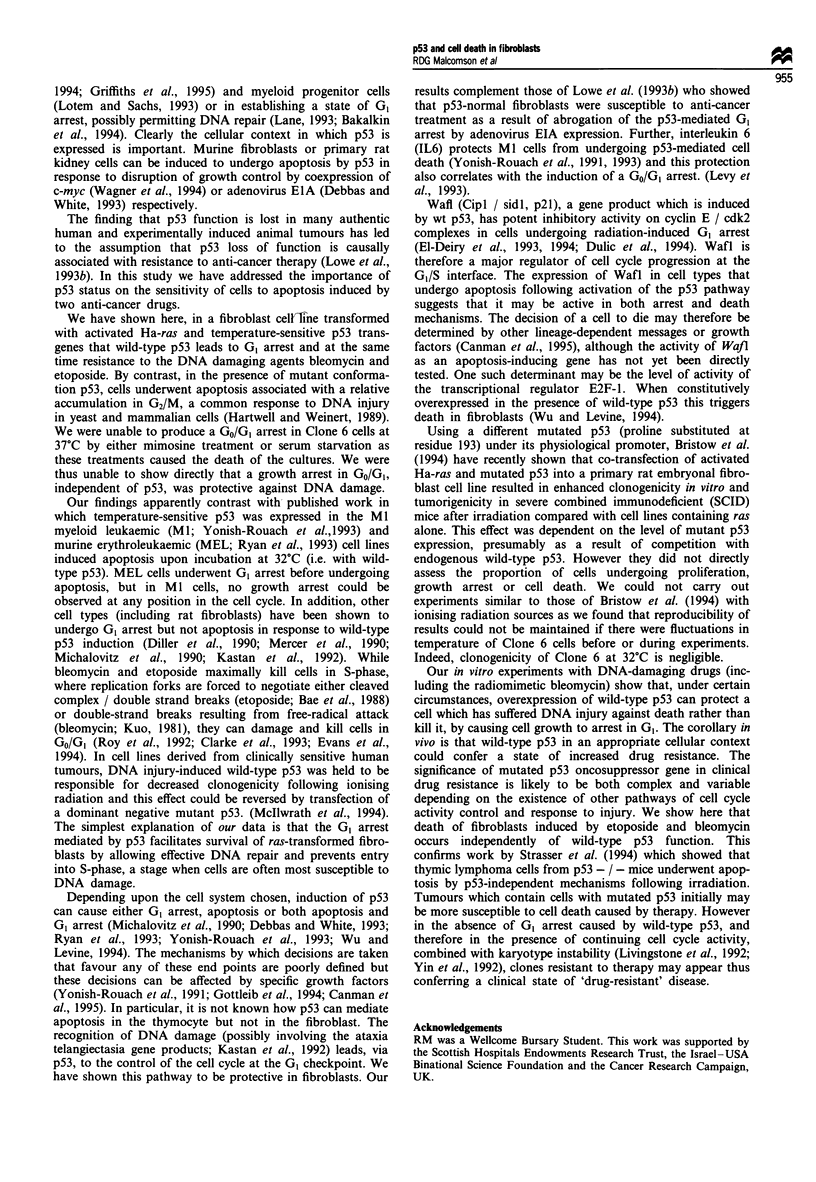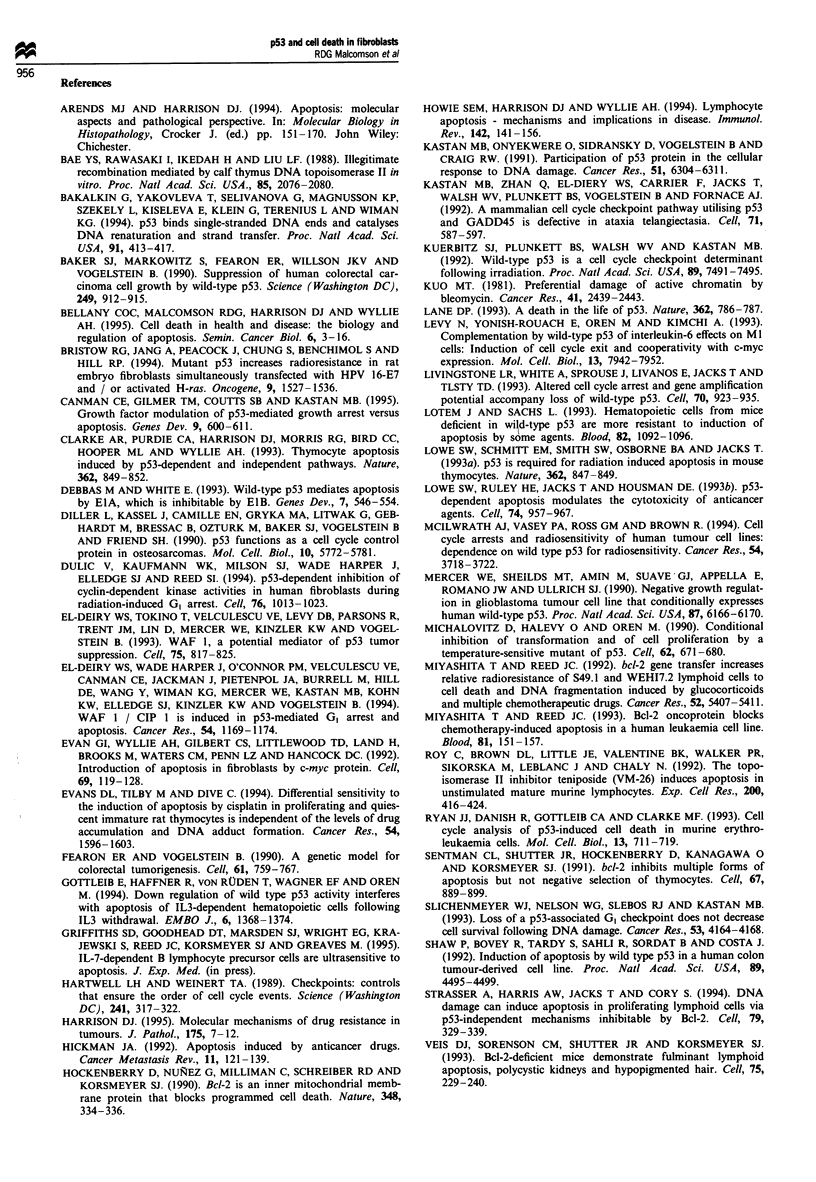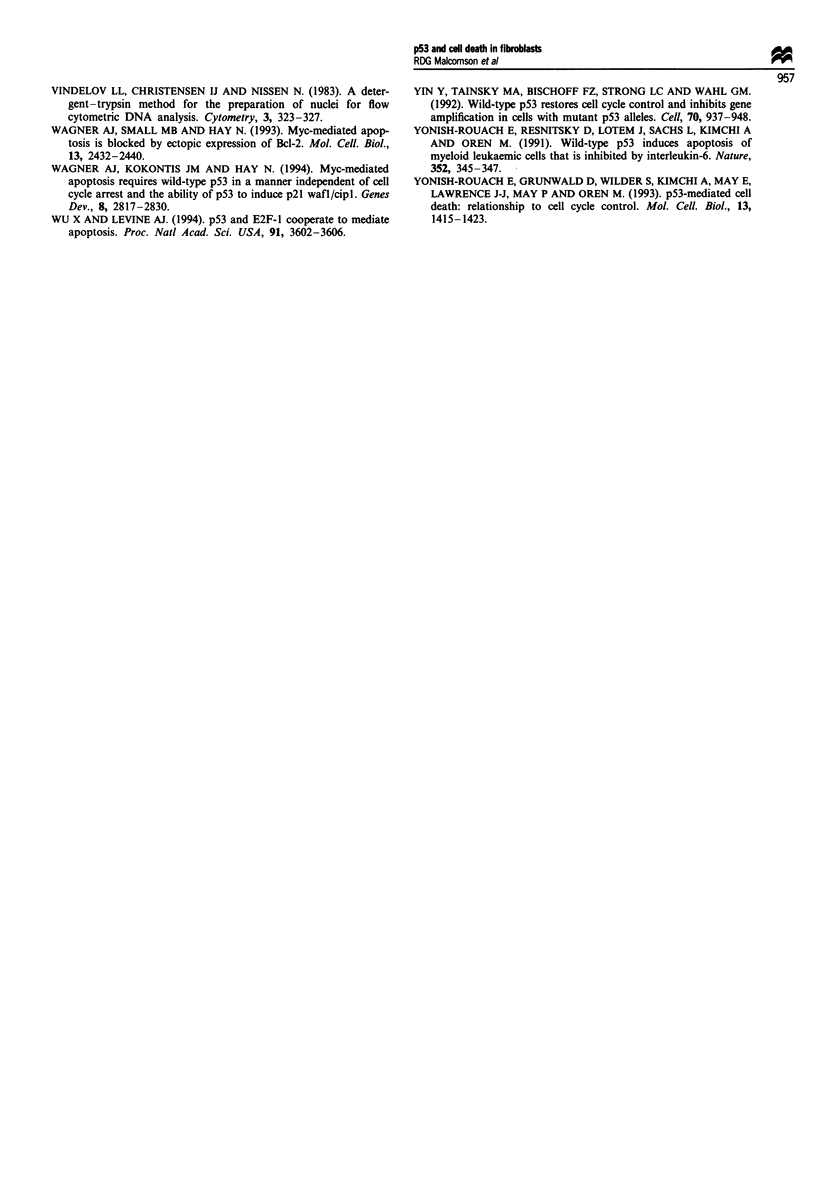# p53-independent death and p53-induced protection against apoptosis in fibroblasts treated with chemotherapeutic drugs.

**DOI:** 10.1038/bjc.1995.440

**Published:** 1995-10

**Authors:** R. D. Malcomson, M. Oren, A. H. Wyllie, D. J. Harrison

**Affiliations:** Department of Pathology, University of Edinburgh Medical School, UK.

## Abstract

Many recent studies have implicated p53 in the cellular response to injury and induction of cell death by apoptosis. In a rat embryonal fibroblast cell line transformed with c-Ha-ras and a mutant temperature-sensitive p53 (val135), cells were G1 arrested at the permissive temperature of 32 degrees C when overexpressed p53 was in wild-type conformation. In this state cells were resistant to apoptosis induced by etoposide (at up to 50 microM) or bleomycin (15 microU ml-1). Cells at 37 degrees C with overexpressed p53 in mutant conformation were freed from this growth arrest, continued proliferating and showed dose-dependent increases in apoptosis. This death is independent of wild-type p53 function. Control cells containing a non-temperature-sensitive mutant p53 (phe132) were sensitive to both etoposide and bleomycin after 24 h at 32 degrees C and 37 degrees C, indicating that the results are not simply due to temperature effects on pharmacokinetics or DNA damage. Our data show that induction of a stable p53-mediated growth arrest renders these cells much less likely to undergo apoptosis in response to certain anti-cancer drugs, and we conclude that the regulatory role of p53 in apoptosis is influenced by the particular cellular context in which this gene is expressed.


British Journal of Cancer (1995) 72, 952-957
© 1995 Stockton Press All rights reserved 0007-0920/95 $12.00

### p53-independent death and p53-induced protection against apoptosis in fibroblasts treated with chemotherapeutic drugs

RDG Malcomson[1], M Oren[2], AH Wyllie[1] and DJ Harrison[1]

[1]*Cancer Research Campaign Laboratories, Department of Pathology, University of Edinburgh Medical School, Teviot Place, Edinburgh EH8 9AG, UK;* [2]*Department of Chemical Immunology, Weizmann Institute of Science, Rehovot 76100, Israel.*

**Summary** Many recent studies have implicated p53 in the cellular response to injury and induction of cell death by apoptosis. In a rat embryonal fibroblast cell line transformed with c-Ha-*ras* and a mutant temperature-sensitive p53 (val135), cells were $G_1$ arrested at the permissive temperature of 32°C when overexpressed p53 was in wild-type conformation. In this state cells were resistant to apoptosis induced by etoposide (at up to 50 μM) or bleomycin (15 μU ml$^{-1}$). Cells at 37°C with overexpressed p53 in mutant conformation were freed from this growth arrest, continued proliferating and showed dose-dependent increases in apoptosis. This death is independent of wild-type p53 function. Control cells containing a non-temperature-sensitive mutant p53 (phe132) were sensitive to both etoposide and bleomycin after 24 h at 32°C and 37°C, indicating that the results are not simply due to temperature effects on pharmacokinetics or DNA damage. Our data show that induction of a stable p53-mediated growth arrest renders these cells much less likely to undergo apoptosis in response to certain anti-cancer drugs, and we conclude that the regulatory role of p53 in apoptosis is influenced by the particular cellular context in which this gene is expressed.

**Keywords:** p53; *ras*; apoptosis; etoposide; bleomycin; cell cycle; fibroblasts

Many tumours are resistant to chemotherapy, either intrinsically or following an initial partial response. A number of pharmacokinetic explanations may account for this, including overexpression of the multidrug resistance gene *mdr*1, overexpression of drug detoxication enzymes, or alteration of the drug target, for example topoisomerase II isoform. However despite intensive study of drug–target interactions, and drug metabolism, it is clear that in many instances drug resistance is associated with a failure of induction of apoptosis, even after an appropriate triggering event. Since many anti-cancer drugs and ionising radiation damage DNA, the response of the cell in recognising injury and proceeding to repair or apoptosis is of paramount importance (Hickman, 1992; Harrison, 1995).

Entry to apoptosis is regulated by a number of genes (see Bellamy *et al.*, 1995 for general review), each of which may show abnormal expression or function in cancer. In Rat-1 fibroblasts cell cycle arrest or serum deprivation in the presence of constitutive expression of the c-*myc* oncogene can cause apoptosis (Evan *et al.*, 1992). By contrast, overexpression of *bcl*-2 directly inhibits apoptosis in both normal and neoplastic cells (Hockenberry *et al.*, 1990; Sentman *et al.*, 1991; Miyashita and Reed, 1992, 1993; Veis *et al.*, 1993) and prevents c-*myc*-driven apoptosis (Wagner *et al.*, 1993). More recently evidence has accumulated implicating the tumour-suppressor gene p53 in an injury-response pathway leading to apoptosis. Thymocytes and myeloid progenitor cells from p53 knockout mice, fail to undergo *induced* apoptosis in the absence of a wild-type p53 allele following etoposide or ionising radiation treatment but not apoptosis associated with ageing *in vitro* or non-clastogenic insults such as dexamethasone treatment. (Clarke *et al.*, 1993; Lotem and Sachs, 1993; Lowe *et al.*, 1993*a*). Furthermore overexpression of wild-type p53 in a variety of cancer-derived cell lines such as M1 myeloid leukaemia (Yonish-Rouach *et al.*, 1991), murine erythroleukaemia (Ryan *et al.*, 1993) and HT29 colon carcinoma (Shaw *et al.*, 1992) resulted in an increase in *spontaneous* apoptosis.

By contrast, studies of p53 null fibroblasts grown in primary culture have failed to detect alteration in cell survival characteristics after DNA damage as compared with normal primary fibroblasts (Slichenmeyer *et al.*, 1993). In the latter experiments, cells were isogenic apart from p53 status. This suggests that other factors, including cell lineage and expression of oncogenes may modulate the effects of p53 on cellular physiology. In both experimental and human tumorigenesis p53 inactivation is believed to be a late event and is therefore superimposed on a series of progressive genetic abnormalities, such as activation of *ras* oncogenes (Fearon and Vogelstein, 1990).

In this study we have used a rat embryonal fibroblast line (Clone 6) transformed with activated Ha-*ras* and a temperature-sensitive p53 mutant as a model of the role of p53 in anti-cancer drug therapy in the presence of other genetic alterations. We report that induction by wild-type p53 of a $G_1$ arrest protects Clone 6 cells from apoptosis caused by the anti-cancer drugs etoposide and bleomycin. Our data imply that wild-type p53 provides a mechanism of resistance of cells to chemotherapy but by allowing continued proliferation p53 mutations may nonetheless contribute to the development of drug resistance.

### Materials and methods

### *Clone 6 cells and RcGphe132.4 cells*

Clone 6 cells are rat embryonic fibroblasts constitutively expressing a human mutationally activated c-Ha-*ras*1 gene and a murine, temperature-sensitive p53 mutant, p53val135. At the permissive temperature of 32°C the p53 protein is found predominantly in wild-type configuration but at 37.5°C it adopts mutant conformation and function (Michalovitz *et al.*, 1990). RcGphe132.4 cells contain a temperature-stable p53phe132 mutation in addition to activated c-Ha-*ras*1. The level of p53 expression in these two cell lines is similar. All manipulation of cell lines and counting was performed at the selected temperature to minimise the risk of inadvertent p53 conformational shifts.

### *Cell culture*

Cells were plated in duplicate flasks at a density of $2 \times 10^4$ cells cm$^{-2}$ in Glasgow modified Eagle's medium (GMEM) supplemented with 10% fetal bovine serum (FBS) and antibiotics.

Correspondence: RDG Malcomson
Received 22 July 1994; revised 23 May 1995; accepted 31 May 1995

*Quantitation of cell number and apoptosis*

Reference points (three per flask) were used to count directly the number of cells in × 100 field using a 10 × 10 graticule. This permitted sequential counts at 20, 28 and 42 h after plating at 32°C or 37°C. Apoptotic cells adherent to the monolayer were counted at each time point as well as cell number. The apoptotic cells were recognised by virtue of their spherical, highly refractile appearance under phase contrast. These cells showed the classical appearances of apoptosis and were confirmed by electron microscopy and acridine orange fluorescence microscopy (Arends and Harrison, 1994).

*Effects of bleomycin and etoposide*

Twenty four hours after plating at 37°C cells were either moved to a 32°C incubator or maintained at 37°C for a further 16 h. Etoposide (10, 50 μM) or bleomycin sulphate (15 μU ml$^{-1}$: 1 U = 1 mg bleomycin A2) were added for 1 h and then washed with phosphate buffered saline (PBS). Controls were performed using equal concentrations of dimethyl sulphoxide (DMSO) or PBS vehicles.

The number of viable and apoptotic cells was counted at intervals up to 50 h following drug treatment. The mean number of apoptotic bodies per field was expressed as a percentage of the mean adherent cell number ('percentage apoptosis'). RcGphe132.4 cells were counted 24 h after drug treatment.

*Cell cycle analysis*

Nuclei were isolated and stained with propidium iodide (Vindelov *et al.*, 1983), and 1 × 10$^4$ cells were analysed on an EPICS CS flow cytometry (Coulter). Histogram analysis was performed using the Easy 2 Software. No doublets were seen. Bromodeoxyuridine incorporation analysis was carried out using the Amersham Cell Proliferation Kit (cat no. RPN20).

### Results

*Clone 6 cells are growth arrested at 32°C*

Exponentially growing cells were shifted to a 32°C incubator. In three independent experiments cells ceased to show increase in cell number at 32°C (Figure 1). There was no increase in apoptosis in the presence of p53 with wild-type configuration (see Figure 2 controls). At 37°C, with mutant conformation p53 there was a 3-fold increase in cell number over the same period, confirming the original observations of Michalovitz *et al.* (1990). DNA flow cytometry showed both diploid and tetraploid peaks at permissive and non-permissive temperatures. At 32°C there was in increase in the diploid $G_{0/1}$ peak (Figure 3), and cells did not take up bromodeoxyuridine consistent with this state (data not shown). This growth arrest was reversible by transferring cells to 37°C, even after 2 weeks, or more. By contrast RcGphe132.4 cells continued to grow at a slightly reduced rate at 32°C in keeping with the previous observations of Michalovitz *et al.* (1990).

*Clone 6 cells with wild type p53 are resistant to both etoposide and bleomycin*

At 37°C, in the presence of mutant conformation p53, there was a progressive increase in apoptosis starting 6–10 h after pulsing with drug (Figure 2). The increase was dose dependent: etoposide at 10 μM induced a maximum of 6% apoptosis whereas at 50 μM the maximum was greater than 30% apoptosis (Figure 4). By contrast, cells maintained at 32°C with p53 in the wild-type conformation showed no increase in percentage apoptosis, nor in cell number (Figures 2 and 4). Treatment with bleomycin showed similar effects (Figure 5).

RcGphe132.4 cells are sensitive to apoptosis induced by etoposide and bleomycin at 32°C and 37°C. We considered the possibility that these differences in cell proliferation and apoptosis in response to DNA damage might be due simply to altered pharmacokinetics at the different temperatures. The RcGphe132.4 cell line was derived from the same parental stock as Clone 6, but contains a temperature-insensitive mutant p53; hence in this cell line wild-type p53 is excluded from function at both 32°C and 37°C. At 37°C Clone 6 and RcGphe132.4 cells show closely similar entry into apoptosis: 24 h after treatment with 50 μM etoposide the incidences were 18.0% and 19.4% respectively. In contrast, at 32°C the incidence of apoptosis in RcGphe132.4 cells was 9.5%, but had fallen to less than 2% in Clone 6 cells. Very similar results were obtained following treatment with bleomycin. At 37°C incidence of apoptosis in Clone 6 cells was 19.8%, but fell to less than 3% at 32°C. In contrast, RcGphe132.4 cells

**Figure 1** Growth properties of Clone 6 cells at 37°C (●) and at 32°C (○). Each point represents the mean number of cells per field ($n$ = 3) in one flask at each time point. Note that the cells incubated at 32°C do not increase in number consistent with a wt p53-induced growth arrest.

**Figure 2** Treatment of Clone 6 cells with 10 μM etoposide at 37°C (●) for 1 h results in substantial apoptosis, where as treated cells at 32°C (■) and untreated controls (unfilled symbols) do not show this increase. Note the latent period during induction of apoptosis at 37°C. Each line represents a separate experiment (performed in triplicate and expressed as a mean, for low values the range was less than 0.6% and for higher value the range was up to 2%).

showed 10.3% apoptosis at 37°C and 8.9% at 32°C. Thus the profound inhibition of apoptosis in Clone 6 cells at 32°C is dependent upon the altered configuration of p53 to wild-type and is not explicable solely on the basis of temperature effects on pharmacokinetics.

### Discussion

Expression of wt p53 has been shown to induce apoptosis in some cell types (Yonish-Rouach *et al.*, 1991; Shaw *et al.*, 1992; Ryan *et al.*, 1993), $G_1$ arrest and survival in others (Baker *et al.*, 1990; Diller *et al.*, 1990; Mercer *et al.*, 1990; Michalovitz *et al.*, 1990; Kastan *et al.*, 1991; Kuerbitz *et al.*, 1992). In addition, wt p53 has been shown to be an essential intermediate in a signal transduction pathway between the effects of DNA damaging agents (DNA strand breaks) and either apoptosis or $G_1$ arrest (Kastan *et al.*, 1992; Kuerbitz *et al.*, 1992; Clarke *et al.*, 1993; Lowe *et al.*, 1993*a*). In this way p53 seems to play a critical role in deleting certain cell types that have sustained DNA damage e.g. thymocytes (Clarke *et al.*, 1993), lymphocytes (Gottlieb *et al.*, 1994; Howie *et al.*,

**Figure 3** Cell cycle analysis of Clone 6 cells. (**a**) Exponentially growing cells at 37°C, untreated ($G_0/G_1$ fraction: 46.56%). (**b**) Following incubation at 32°C for 24 h, the $G_0/G_1$ peak is enlarged (72.51%) and there is a marked decrease in the proportion of cells between the $G_0/G_1$ and $G_2/M$ peaks (S-phase). (**c**) At 37°C, 24 h after etoposide treatment (50 $\mu$M) cells accumulated in $G_2/M$ with only 10.18% of cells occupying the $G_0/G_1$ position. (**d**) At 32°C, 24 h after treatment with 50 $\mu$M etoposide ($G_0/G_1$ fraction: 38.51%). Abcissa; DNA content (propidium iodide fluorescence).

**Figure 4** Treatment of Clone 6 cells with 50 $\mu$M etoposide for 1 h induces substantial apoptosis when cells are incubated at 37°C (●). At 32°C treated cells (■) and in control cells treated with an equivalent volume of DMSO vehicle (unfilled symbols) do not show an increase in percentage apoptosis. Each line represents a separate experiment (performed in triplicate and expressed as a mean. For low values range was 2% and for high values was up to 22%).

**Figure 5** Clone 6 cells treated with 15 $\mu$U $ml^{-1}$ bleomycin sulphate for 1 h at 37°C (●) and at 32°C (■). Note that treated cells incubated at 37°C undergo substantial apoptosis whereas treated cells at 32°C and untreated controls (open symbols) do not shown an increase in percentage apoptosis. Each line represents a separate experiment (performed in triplicate and expressed as a mean. For low values range was 2% and for high values range was up to 12%).

1994; Griffiths *et al.*, 1995) and myeloid progenitor cells (Lotem and Sachs, 1993) or in establishing a state of $G_1$ arrest, possibly permitting DNA repair (Lane, 1993; Bakalkin *et al.*, 1994). Clearly the cellular context in which p53 is expressed is important. Murine fibroblasts or primary rat kidney cells can be induced to undergo apoptosis by p53 in response to disruption of growth control by coexpression of c-*myc* (Wagner *et al.*, 1994) or adenovirus E1A (Debbas and White, 1993) respectively.

The finding that p53 function is lost in many authentic human and experimentally induced animal tumours has led to the assumption that p53 loss of function is causally associated with resistance to anti-cancer therapy (Lowe *et al.*, 1993*b*). In this study we have addressed the importance of p53 status on the sensitivity of cells to apoptosis induced by two anti-cancer drugs.

We have shown here, in a fibroblast cell line transformed with activated Ha-*ras* and temperature-sensitive p53 transgenes that wild-type p53 leads to $G_1$ arrest and at the same time resistance to the DNA damaging agents bleomycin and etoposide. By contrast, in the presence of mutant conformation p53, cells underwent apoptosis associated with a relative accumulation in $G_2$/M, a common response to DNA injury in yeast and mammalian cells (Hartwell and Weinert, 1989). We were unable to produce a $G_0/G_1$ arrest in Clone 6 cells at 37°C by either mimosine treatment or serum starvation as these treatments caused the death of the cultures. We were thus unable to show directly that a growth arrest in $G_0/G_1$, independent of p53, was protective against DNA damage.

Our findings apparently contrast with published work in which temperature-sensitive p53 was expressed in the M1 myeloid leukaemic (M1; Yonish-Rouach *et al.*,1993) and murine erythroleukaemic (MEL; Ryan *et al.*, 1993) cell lines induced apoptosis upon incubation at 32°C (i.e. with wild-type p53). MEL cells underwent $G_1$ arrest before undergoing apoptosis, but in M1 cells, no growth arrest could be observed at any position in the cell cycle. In addition, other cell types (including rat fibroblasts) have been shown to undergo $G_1$ arrest but not apoptosis in response to wild-type p53 induction (Diller *et al.*, 1990; Mercer *et al.*, 1990; Michalovitz *et al.*, 1990; Kastan *et al.*, 1992). While bleomycin and etoposide maximally kill cells in S-phase, where replication forks are forced to negotiate either cleaved complex / double strand breaks (etoposide; Bae *et al.*, 1988) or double-strand breaks resulting from free-radical attack (bleomycin; Kuo, 1981), they can damage and kill cells in $G_0/G_1$ (Roy *et al.*, 1992; Clarke *et al.*, 1993; Evans *et al.*, 1994). In cell lines derived from clinically sensitive human tumours, DNA injury-induced wild-type p53 was held to be responsible for decreased clonogenicity following ionising radiation and this effect could be reversed by transfection of a dominant negative mutant p53. (McIlwrath *et al.*, 1994). The simplest explanation of *our* data is that the $G_1$ arrest mediated by p53 facilitates survival of *ras*-transformed fibroblasts by allowing effective DNA repair and prevents entry into S-phase, a stage when cells are often most susceptible to DNA damage.

Depending upon the cell system chosen, induction of p53 can cause either $G_1$ arrest, apoptosis or both apoptosis and $G_1$ arrest (Michalovitz *et al.*, 1990; Debbas and White, 1993; Ryan *et al.*, 1993; Yonish-Rouach *et al.*, 1993; Wu and Levine, 1994). The mechanisms by which decisions are taken that favour any of these end points are poorly defined but these decisions can be affected by specific growth factors (Yonish-Rouach *et al.*, 1991; Gottleib *et al.*, 1994; Canman *et al.*, 1995). In particular, it is not known how p53 can mediate apoptosis in the thymocyte but not in the fibroblast. The recognition of DNA damage (possibly involving the ataxia telangiectasia gene products; Kastan *et al.*, 1992) leads, via p53, to the control of the cell cycle at the $G_1$ checkpoint. We have shown this pathway to be protective in fibroblasts. Our results complement those of Lowe *et al.* (1993*b*) who showed that p53-normal fibroblasts were susceptible to anti-cancer treatment as a result of abrogation of the p53-mediated $G_1$ arrest by adenovirus EIA expression. Further, interleukin 6 (IL6) protects M1 cells from undergoing p53-mediated cell death (Yonish-Rouach *et al.*, 1991, 1993) and this protection also correlates with the induction of a $G_0/G_1$ arrest. (Levy *et al.*, 1993).

Wafl (Cipl / sidl, p21), a gene product which is induced by wt p53, has potent inhibitory activity on cyclin E / cdk2 complexes in cells undergoing radiation-induced $G_1$ arrest (El-Deiry *et al.*, 1993, 1994; Dulic *et al.*, 1994). Wafl is therefore a major regulator of cell cycle progression at the $G_1$/S interface. The expression of Wafl in cell types that undergo apoptosis following activation of the p53 pathway suggests that it may be active in both arrest and death mechanisms. The decision of a cell to die may therefore be determined by other lineage-dependent messages or growth factors (Canman *et al.*, 1995), although the activity of *Wafl* as an apoptosis-inducing gene has not yet been directly tested. One such determinant may be the level of activity of the transcriptional regulator E2F-1. When constitutively overexpressed in the presence of wild-type p53 this triggers death in fibroblasts (Wu and Levine, 1994).

Using a different mutated p53 (proline substituted at residue 193) under its physiological promoter, Bristow *et al.* (1994) have recently shown that co-transfection of activated Ha-*ras* and mutated p53 into a primary rat embryonal fibroblast cell line resulted in enhanced clonogenicity *in vitro* and tumorigenicity in severe combined immunodeficient (SCID) mice after irradiation compared with cell lines containing *ras* alone. This effect was dependent on the level of mutant p53 expression, presumably as a result of competition with endogenous wild-type p53. However they did not directly assess the proportion of cells undergoing proliferation, growth arrest or cell death. We could not carry out experiments similar to those of Bristow *et al.* (1994) with ionising radiation sources as we found that reproducibility of results could not be maintained if there were fluctuations in temperature of Clone 6 cells before or during experiments. Indeed, clonogenicity of Clone 6 at 32°C is negligible.

Our *in vitro* experiments with DNA-damaging drugs (including the radiomimetic bleomycin) show that, under certain circumstances, overexpression of wild-type p53 can protect a cell which has suffered DNA injury against death rather than kill it, by causing cell growth to arrest in $G_1$. The corollary *in vivo* is that wild-type p53 in an appropriate cellular context could confer a state of increased drug resistance. The significance of mutated p53 oncosuppressor gene in clinical drug resistance is likely to be both complex and variable depending on the existence of other pathways of cell cycle activity control and response to injury. We show here that death of fibroblasts induced by etoposide and bleomycin occurs independently of wild-type p53 function. This confirms work by Strasser *et al.* (1994) which showed that thymic lymphoma cells from p53 − / − mice underwent apoptosis by p53-independent mechanisms following irradiation. Tumours which contain cells with mutated p53 initially may be more susceptible to cell death caused by therapy. However in the absence of $G_1$ arrest caused by wild-type p53, and therefore in the presence of continuing cell cycle activity, combined with karyotype instability (Livingstone *et al.*, 1992; Yin *et al.*, 1992), clones resistant to therapy may appear thus conferring a clinical state of 'drug-resistant' disease.

**Acknowledgements**
RM was a Wellcome Bursary Student. This work was supported by the Scottish Hospitals Endowments Research Trust, the Israel-USA Binational Science Foundation and the Cancer Research Campaign, UK.

### References

ARENDS MJ AND HARRISON DJ. (1994). Apoptosis: molecular aspects and pathological perspective. In: *Molecular Biology in Histopathology*, Crocker J. (ed.) pp. 151–170. John Wiley: Chichester.

BAE YS, RAWASAKI I, IKEDAH H AND LIU LF. (1988). Illegitimate recombination mediated by calf thymus DNA topoisomerase II *in vitro*. *Proc. Natl Acad. Sci. USA.*, **85**, 2076–2080.

BAKALKIN G, YAKOVLEVA T, SELIVANOVA G, MAGNUSSON KP, SZEKELY L, KISELEVA E, KLEIN G, TERENIUS L AND WIMAN KG. (1994). p53 binds single-stranded DNA ends and catalyses DNA renaturation and strand transfer. *Proc. Natl Acad. Sci. USA*, **91**, 413–417.

BAKER SJ, MARKOWITZ S, FEARON ER, WILLSON JKV AND VOGELSTEIN B. (1990). Suppression of human colorectal carcinoma cell growth by wild-type p53. *Science (Washington DC)*, **249**, 912–915.

BELLANY COC, MALCOMSON RDG, HARRISON DJ AND WYLLIE AH. (1995). Cell death in health and disease: the biology and regulation of apoptosis. *Semin. Cancer Biol.* **6**, 3–16.

BRISTOW RG, JANG A, PEACOCK J, CHUNG S, BENCHIMOL S AND HILL RP. (1994). Mutant p53 increases radioresistance in rat embryo fibroblasts simultaneously transfected with HPV 16-E7 and / or activated H-*ras*. *Oncogene*, **9**, 1527–1536.

CANMAN CE, GILMER TM, COUTTS SB AND KASTAN MB. (1995). Growth factor modulation of p53-mediated growth arrest versus apoptosis. *Genes Dev.* **9**, 600–611.

CLARKE AR, PURDIE CA, HARRISON DJ, MORRIS RG, BIRD CC, HOOPER ML AND WYLLIE AH. (1993). Thymocyte apoptosis induced by p53-dependent and independent pathways. *Nature*, **362**, 849–852.

DEBBAS M AND WHITE E. (1993). Wild-type p53 mediates apoptosis by E1A, which is inhibitable by E1B. *Genes Dev.*, **7**, 546–554.

DILLER L, KASSEL J, CAMILLE EN, GRYKA MA, LITWAK G, GEBHARDT M, BRESSAC B, OZTURK M, BAKER SJ, VOGELSTEIN B AND FRIEND SH. (1990). p53 functions as a cell cycle control protein in osteosarcomas. *Mol. Cell. Biol.*, **10**, 5772–5781.

DULIC V, KAUFMANN WK, MILSON SJ, WADE HARPER J, ELLEDGE SJ AND REED SI. (1994). p53-dependent inhibition of cyclin-dependent kinase activities in human fibroblasts during radiation-induced $G_1$ arrest. *Cell*, **76**, 1013–1023.

EL-DEIRY WS, TOKINO T, VELCULESCU VE, LEVY DB, PARSONS R, TRENT JM, LIN D, MERCER WE, KINZLER KW AND VOGELSTEIN B. (1993). WAF 1, a potential mediator of p53 tumor suppression. *Cell*, **75**, 817–825.

EL-DEIRY WS, WADE HARPER J, O'CONNOR PM, VELCULESCU VE, CANMAN CE, JACKMAN J, PIETENPOL JA, BURRELL M, HILL DE, WANG Y, WIMAN KG, MERCER WE, KASTAN MB, KOHN KW, ELLEDGE SJ, KINZLER KW AND VOGELSTEIN B. (1994). WAF 1 / CIP 1 is induced in p53-mediated $G_1$ arrest and apoptosis. *Cancer Res.*, **54**, 1169–1174.

EVAN GI, WYLLIE AH, GILBERT CS, LITTLEWOOD TD, LAND H, BROOKS M, WATERS CM, PENN LZ AND HANCOCK DC. (1992). Introduction of apoptosis in fibroblasts by c-*myc* protein. *Cell*, **69**, 119–128.

EVANS DL, TILBY M AND DIVE C. (1994). Differential sensitivity to the induction of apoptosis by cisplatin in proliferating and quiescent immature rat thymocytes is independent of the levels of drug accumulation and DNA adduct formation. *Cancer Res.*, **54**, 1596–1603.

FEARON ER AND VOGELSTEIN B. (1990). A genetic model for colorectal tumorigenesis. *Cell*, **61**, 759–767.

GOTTLEIB E, HAFFNER R, VON RÜDEN T, WAGNER EF AND OREN M. (1994). Down regulation of wild type p53 activity interferes with apoptosis of IL3-dependent hematopoietic cells following IL3 withdrawal. *EMBO J.*, **6**, 1368–1374.

GRIFFITHS SD, GOODHEAD DT, MARSDEN SJ, WRIGHT EG, KRAJEWSKI S, REED JC, KORSMEYER SJ AND GREAVES M. (1995). IL-7-dependent B lymphocyte precursor cells are ultrasensitive to apoptosis. *J. Exp. Med.* (in press).

HARTWELL LH AND WEINERT TA. (1989). Checkpoints: controls that ensure the order of cell cycle events. *Science (Washington DC)*, **241**, 317–322.

HARRISON DJ. (1995). Molecular mechanisms of drug resistance in tumours. *J. Pathol.*, **175**, 7–12.

HICKMAN JA. (1992). Apoptosis induced by anticancer drugs. *Cancer Metastasis Rev.*, **11**, 121–139.

HOCKENBERRY D, NUÑEZ G, MILLIMAN C, SCHREIBER RD AND KORSMEYER SJ. (1990). *Bcl*-2 is an inner mitochondrial membrane protein that blocks programmed cell death. *Nature*, **348**, 334–336.

HOWIE SEM, HARRISON DJ AND WYLLIE AH. (1994). Lymphocyte apoptosis - mechanisms and implications in disease. *Immunol. Rev.*, **142**, 141–156.

KASTAN MB, ONYEKWERE O, SIDRANSKY D, VOGELSTEIN B AND CRAIG RW. (1991). Participation of p53 protein in the cellular response to DNA damage. *Cancer Res.*, **51**, 6304–6311.

KASTAN MB, ZHAN Q, EL-DIERY WS, CARRIER F, JACKS T, WALSH WV, PLUNKETT BS, VOGELSTEIN B AND FORNACE AJ. (1992). A mammalian cell cycle checkpoint pathway utilising p53 and GADD45 is defective in ataxia telangiectasia. *Cell*, **71**, 587–597.

KUERBITZ SJ, PLUNKETT BS, WALSH WV AND KASTAN MB. (1992). Wild-type p53 is a cell cycle checkpoint determinant following irradiation. *Proc. Natl Acad. Sci. USA*, **89**, 7491–7495.

KUO MT. (1981). Preferential damage of active chromatin by bleomycin. *Cancer Res.*, **41**, 2439–2443.

LANE DP. (1993). A death in the life of p53. *Nature*, **362**, 786–787.

LEVY N, YONISH-ROUACH E, OREN M AND KIMCHI A. (1993). Complementation by wild-type p53 of interleukin-6 effects on M1 cells: Induction of cell cycle exit and cooperativity with c-myc expression. *Mol. Cell. Biol.*, **13**, 7942–7952.

LIVINGSTONE LR, WHITE A, SPROUSE J, LIVANOS E, JACKS T AND TLSTY TD. (1993). Altered cell cycle arrest and gene amplification potential accompany loss of wild-type p53. *Cell*, **70**, 923–935.

LOTEM J AND SACHS L. (1993). Hematopoietic cells from mice deficient in wild-type p53 are more resistant to induction of apoptosis by some agents. *Blood*, **82**, 1092–1096.

LOWE SW, SCHMITT EM, SMITH SW, OSBORNE BA AND JACKS T. (1993*a*). p53 is required for radiation induced apoptosis in mouse thymocytes. *Nature*, **362**, 847–849.

LOWE SW, RULEY HE, JACKS T AND HOUSMAN DE. (1993*b*). p53-dependent apoptosis modulates the cytotoxicity of anticancer agents. *Cell*, **74**, 957–967.

MCILWRATH AJ, VASEY PA, ROSS GM AND BROWN R. (1994). Cell cycle arrests and radiosensitivity of human tumour cell lines: dependence on wild type p53 for radiosensitivity. *Cancer Res.*, **54**, 3718–3722.

MERCER WE, SHEILDS MT, AMIN M, SUAVE GJ, APPELLA E, ROMANO JW AND ULLRICH SJ. (1990). Negative growth regulation in glioblastoma tumour cell line that conditionally expresses human wild-type p53. *Proc. Natl Acad. Sci. USA*, **87**, 6166–6170.

MICHALOVITZ D, HALEVY O AND OREN M. (1990). Conditional inhibition of transformation and of cell proliferation by a temperature-sensitive mutant of p53. *Cell*, **62**, 671–680.

MIYASHITA T AND REED JC. (1992). *bcl*-2 gene transfer increases relative radioresistance of S49.1 and WEHI7.2 lymphoid cells to cell death and DNA fragmentation induced by glucocorticoids and multiple chemotherapeutic drugs. *Cancer Res.*, **52**, 5407–5411.

MIYASHITA T AND REED JC. (1993). Bcl-2 oncoprotein blocks chemotherapy-induced apoptosis in a human leukaemia cell line. *Blood*, **81**, 151–157.

ROY C, BROWN DL, LITTLE JE, VALENTINE BK, WALKER PR, SIKORSKA M, LEBLANC J AND CHALY N. (1992). The topoisomerase II inhibitor teniposide (VM-26) induces apoptosis in unstimulated mature murine lymphocytes. *Exp. Cell Res.*, **200**, 416–424.

RYAN JJ, DANISH R, GOTTLEIB CA AND CLARKE MF. (1993). Cell cycle analysis of p53-induced cell death in murine erythroleukaemia cells. *Mol. Cell. Biol.*, **13**, 711–719.

SENTMAN CL, SHUTTER JR, HOCKENBERRY D, KANAGAWA O AND KORSMEYER SJ. (1991). *bcl*-2 inhibits multiple forms of apoptosis but not negative selection of thymocytes. *Cell*, **67**, 889–899.

SLICHENMEYER WJ, NELSON WG, SLEBOS RJ AND KASTAN MB. (1993). Loss of a p53-associated $G_1$ checkpoint does not decrease cell survival following DNA damage. *Cancer Res.*, **53**, 4164–4168.

SHAW P, BOVEY R, TARDY S, SAHLI R, SORDAT B AND COSTA J. (1992). Induction of apoptosis by wild type p53 in a human colon tumour-derived cell line. *Proc. Natl Acad. Sci. USA*, **89**, 4495–4499.

STRASSER A, HARRIS AW, JACKS T AND CORY S. (1994). DNA damage can induce apoptosis in proliferating lymphoid cells via p53-independent mechanisms inhibitable by Bcl-2. *Cell*, **79**, 329–339.

VEIS DJ, SORENSON CM, SHUTTER JR AND KORSMEYER SJ. (1993). Bcl-2-deficient mice demonstrate fulminant lymphoid apoptosis, polycystic kidneys and hypopigmented hair. *Cell*, **75**, 229–240.

VINDELOV LL, CHRISTENSEN IJ AND NISSEN N. (1983). A detergent–trypsin method for the preparation of nuclei for flow cytometric DNA analysis. *Cytometry*, **3**, 323–327.

WAGNER AJ, SMALL MB AND HAY N. (1993). Myc-mediated apoptosis is blocked by ectopic expression of Bcl-2. *Mol. Cell. Biol.*, **13**, 2432–2440.

WAGNER AJ, KOKONTIS JM AND HAY N. (1994). Myc-mediated apoptosis requires wild-type p53 in a manner independent of cell cycle arrest and the ability of p53 to induce p21 waf1/cip1. *Genes Dev.*, **8**, 2817–2830.

WU X AND LEVINE AJ. (1994). p53 and E2F-1 cooperate to mediate apoptosis. *Proc. Natl Acad. Sci. USA*, **91**, 3602–3606.

YIN Y, TAINSKY MA, BISCHOFF FZ, STRONG LC AND WAHL GM. (1992). Wild-type p53 restores cell cycle control and inhibits gene amplification in cells with mutant p53 alleles. *Cell*, **70**, 937–948.

YONISH-ROUACH E, RESNITSKY D, LOTEM J, SACHS L, KIMCHI A AND OREN M. (1991). Wild-type p53 induces apoptosis of myeloid leukaemic cells that is inhibited by interleukin-6. *Nature*, **352**, 345–347.

YONISH-ROUACH E, GRUNWALD D, WILDER S, KIMCHI A, MAY E, LAWRENCE J-J, MAY P AND OREN M. (1993). p53-mediated cell death: relationship to cell cycle control. *Mol. Cell. Biol.*, **13**, 1415–1423.